# The Effect of Fibroblast Growth Factor 15 Signaling in Non-Steatotic and Steatotic Liver Transplantation from Cardiocirculatory Death

**DOI:** 10.3390/cells8121640

**Published:** 2019-12-14

**Authors:** Cindy G. Avalos-de León, Mónica B. Jiménez-Castro, María Eugenia Cornide-Petronio, José Gulfo, Floriana Rotondo, Jordi Gracia-Sancho, Araní Casillas-Ramírez, Carmen Peralta

**Affiliations:** 1 Institut d’Investigacions Biomèdiques August Pi I Sunyer (IDIBAPS), 08036 Barcelona, Spain; avalosdl.cin@gmail.com (C.G.A.-d.L.); monicabjimenez@hotmail.com (M.B.J.-C.); cornide@clinic.cat (M.E.C.-P.); gulfo@clinic.cat (J.G.); floriana.rotondo@gmail.com (F.R.); 2 Centro de Investigación Biomédica en Red de Enfermedades Hepáticas y Digestivas (CIBERehd), 08036 Barcelona, Spain; jordi.gracia@idibaps.org; 3 Liver Vascular Biology Research Group, IDIBAPS, 08036 Barcelona, Spain; 4 Hospital Regional de Alta Especialidad de Ciudad Victoria “Bicentenario 2010”, Ciudad Victoria 87087, Mexico; 5 Facultad de Medicina e Ingeniería en Sistemas Computacionales de Matamoros, Universidad Autónoma de Tamaulipas, Matamoros 87300, Mexico

**Keywords:** cardiocirculatory death, FGF15, liver transplantation, steatotic liver grafts, ischemia-reperfusion damage

## Abstract

We elucidate the relevance of fibroblast growth factor 15 (FGF15) in liver transplantation (LT) using rats with both steatotic and non-steatotic organs from donors after cardiocirculatory death (DCD). Compared to LT from non-DCDs, the induction of cardiocirculatory death (CD) increases hepatic damage, proliferation, and intestinal and circulatory FGF15. This is associated with high levels of FGF15, bilirubin and bile acids (BAs), and overexpression of the enzyme involved in the alternative BA synthesis pathway, CYP27A1, in non-steatotic livers. Furthermore, CD activates the proliferative pathway, Hippo/YAP, in these types of liver. Blocking FGF15 action in LT from DCDs does not affect CYP27A1 but causes an overexpression of CYP7A, an enzyme from the classic BA synthesis pathway, and this is related to further accumulation of BAs and exacerbated damage. FGF15 inhibition also impairs proliferation without changing Hippo/YAP. In spite of worse damage, steatosis prevents a proliferative response in livers from DCDs. In steatotic grafts, CD does not modify CYP7A1, CYP27A1, BA, or the Hippo/YAP pathway, and FGF15 is not involved in damage or proliferation. Thus, endogenous FGF15 protects against BA accumulation and damage and promotes regeneration independently of the Hippo/YAP pathway, in non-steatotic LT from DCDs. Herein we show a minor role of FGF15 in steatotic LT from DCDs.

## 1. Introduction

Every year, many thousands of deaths result from liver failure that could not be treated due to the lack of suitable donor organs [1]. To better meet clinical demand, the use of livers from donors after cardiocirculatory death (DCDs) is being explored. However, the high incidence of hepatic dysfunction and biliary complications caused by disruption of the blood supply during the donor warm ischemia time after cardiocirculatory death (CD) hinders the utilization of these grafts to liver transplantation (LT) [2,3,4]. In addition, the use of organs from extended criteria donors has increased, including those with hepatic steatosis, due to its high worldwide prevalence [5]. Unfortunately, hepatic steatosis is one of the most important risk factors that negatively affect post-operative outcomes [6].

Fibroblast growth factor 15 (FGF15), along with its human homolog (FGF19), collectively referred to as FGF15/19, regulates bile acids (BAs) [7]. In brief, the ileal enterocytes induce the expression of FGF15/19, which is secreted into the enterohepatic circulation [8]. Through the hepatic fibroblast growth factor receptor 4 (FGFR4) signaling pathway, BA levels are tightly controlled by the enzyme cytochrome P450 7A1 (CYP7A1), the first and rate-limiting step in the classic BA synthesis pathway [9]. In addition to maintaining BA homeostasis, FGF15 regulates liver regeneration through Hippo/Yes-associated protein (YAP) signaling [10,11]. Indeed, the mammalian sterile-20 kinase 1/2 (MST1/2) activates the large tumor suppressor 1/2 (LATS1/2), that phosphorylates YAP [12]. Phospho-YAP is either degraded or sequestered in the cytoplasm [13]. When the Hippo pathway is off, YAP translocates to the nucleus and turns on pro-proliferative and pro-survival genes, thereby enabling cell proliferation [14].

Changes in circulating FGF19 have been reported in patients with liver diseases, such as cirrhosis [15], and in pathologies associated with intestinal inflammation, such as ileal dysfunction due to Crohn’s disease [16]. Several lines of evidence demonstrate that various liver diseases, such as cirrhosis and bile duct ligation, alter BA synthesis by up-regulating the expression of CYP7A1 in the liver [17]. In addition, YAP mediates cell proliferation and regulated cell death and participates in the restoration of the heart after ischemia-reperfusion (I/R) [18]. Along the same lines, ablating YAP in the mouse liver enhances hepatocyte necrosis and suppresses hepatocyte proliferation after bile duct ligation [14].

This is the first study to analyze the effect of FGF15 derived from intestinal signaling in both steatotic and non-steatotic LT from DCDs. Understanding the essential components of this response may facilitate the development of hepatoprotective and pro-regenerative therapeutic strategies in steatotic and non-steatotic LT from DCDs.

## 2. Materials and Methods

### 2.1. Experimental Animals

This study was performed using homozygous (obese, Ob) and heterozygous (lean, Ln) male Zucker rats (Iffa Credo, France) aged between 10 and 11 weeks. Ob rats show moderate macrovesicular and microvesicular fatty infiltration in hepatocytes (40% to 60% steatosis), whereas Ln rats show no evidence of steatosis [19]. All procedures were approved by the Laboratory Animal Care and Use Committee of Barcelona University and by the government of Catalonia (DAAM 9353). European Union regulations (Directive 86/609 EEC) for animal experiments were respected.

### 2.2. Cardiocirculatory Death Induction

Animals were anesthetized by isoflurane and heparin sodium solution was administered to donors. A well-established rodent CD model was selected: induction of CD by hypoxia through an incision of the diaphragm and blocking the descending aorta [20,21]. The in situ warm ischemia period was 45 min.

### 2.3. Surgical Procedure of Liver Transplantation

Steatotic and non-steatotic liver grafts from DCDs and non-DCDs were flushed and stored in University of Wisconsin (UW) solution for 6 h. Standard orthotopic LT in Ln Zucker rats was performed according to the double cuff technique [19].

### 2.4. Experimental Design

Group 1. Sham (n = 12). Six Ob and six Ln Zucker rats were anesthetized and maintained for 45 min.

Group 2. LT (n = 24, 12 transplantations). Six Ob and six Ln Zucker rats were anesthetized and maintained for 45 min. Then, steatotic and non-steatotic liver grafts were flushed, isolated, and stored in ice-cold UW solution for 6 h. Finally, they were implanted in 12 Ln Zucker rats [19].

Group 3. CD + LT (n = 24, 12 transplantations). Six Ob and six Ln Zucker rats were anesthetized. After the induction of CD, the animals were maintained in situ in warm ischemia for 45 min. Then, steatotic and non-steatotic liver grafts were flushed, isolated, and stored in ice-cold UW solution for 6 h. Finally, they were implanted in 12 Ln Zucker rats [19,20,21].

Group 4. CD + FGF15inh + LT (n = 24, 12 transplantations). Procedures as per Group 3 except donors were treated with intravenous FGF401 at a dosage of 200 µg/kg, 10 min before CD induction. FGF401 is a highly selective and potent FGFR4 inhibitor aimed at blocking the actions of FGF15 [20,21,22,23].

Group 5. FGF15inh + LT (n = 24, 12 transplantations). Procedures as per Group 2 except donors were treated with intravenous FGF401 at a dosage of 200 µg/kg, 10 min before surgical procedure [19,23].

Group 6. CD (n = 12). Six Ob and six Ln Zucker rats were anesthetized. After CD induction, rats were maintained in situ in warm ischemia for 45 min [20,21].

Group 7. CD + FGF15inh (n = 12). Procedures as per Group 5 except donors were treated with intravenous FGF401 at a dosage of 200 µg/kg, 10 min before CD induction [20,21,22,23].

Plasma, liver, and intestine samples were collected 4 h after LT (Groups 2–5) as well as before the procurement of liver grafts from donors (Groups 6 and 7). For the current study, conditions, drug dosage, and pre-treatment times were selected based on previous studies [19,20,21,22,23] as well as on preliminary studies by our group.

### 2.5. Biochemical Assays

Plasma transaminases (aspartate aminotransferase (AST) and alanine aminotransferase (ALT)) were measured photometrically using standard procedures. Total bilirubin in plasma was determined by an enzyme-linked immune absorbent assay (MyBiosourse Inc., San Diego, CA, USA) according to the manufacturer’s instructions. Plasma and liver total BAs were determined by a colorimetric assay kit (Biovision, Milpitas, CA, USA).

Lipid peroxidation in the intestine was determined by measuring the formation of malondialdehyde (MDA) as an indirect index of the oxidative injury induced by the reactive oxygen species [24]. Briefly, 0.5 mL of 0.5% butylated hydroxytoluene was added to 2 mL of the intestine homogenate to prevent lipid autoxidation. For protein precipitation, 2 mL of 20% trichloroacetic acid was added to 2 mL of homogenate. After mixing and centrifuging, 1 mL of 0.67% thiobarbiturate solution was added to the supernatant and boiled for 60 min. After cooling, optical density at 530 nm was assayed [24].

Myeloperoxidase (MPO), was measured photometrically as an index of neutrophil accumulation, using tetramethyl-benzidine as a substrate. Intestine samples were macerated with 0.5% hexadecyltrimethylammonium bromide in 50 mM phosphate buffer pH 6.0. Homogenates were then disrupted for 30 s using a sonicator at 20% power and subsequently snap-frozen in dry ice and thawed on three consecutive occasions before a final 30-s sonication. Samples were incubated at 60 °C for 2 h and then spun down at 4000× *g* for 12 min. Supernatants were collected for the MPO assay. Enzyme activity was assessed photometrically at 630 nm. The assay mixture consisted of 20 μL supernatant, 10 μL tetramethylbenzidine (final concentration 1.6 mM) dissolved in dimethyl sulfoxide, and 70 μL H_2_O_2_ (final concentration, 3.0 mM) diluted in 80 Mm phosphate buffer, pH 5.4. An enzyme unit is defined as the amount of enzyme that produces an increase of 1 absorbance unit per minute [25].

### 2.6. Western Blotting

Liver and intestine tissues were homogenized in a lysis buffer, 150 mM NaCl, 50 mM Tris pH 7.5, 1 mM EDTA, 0.5% Triton X-100, 0.5% Nonidet P-40, with protease and phosphatase inhibitors (Sigma Aldrich, St Louis, MO, USA). Samples were sonicated at 60 w for 15 s and then centrifuged at 20,000× *g* for 10 min. Plasma, liver, and intestine homogenates containing an equal amount of protein were mixed in Laemmli loading buffer and were separated on a sodium dodecyl sulfate (SDS-PAGE) 8–12% poly-acrylamide gel electrophoresis and transferred to polyvinylidene fluoride membranes. After assessing transfer, the membranes were saturated in 4 mM Tris–HCl, pH 7.6, 30 mM NaCl (TBST) containing 5% non-fat milk and 0.1% Tween-20 and incubated over-night at 4 °C, using antibodies against the following proteins: FGF15 (LS-B15011) (Life-Span BioSciences Inc., Seattle, Washington, USA); CYP7A1 (ab65596) (Abcam, Cambridge, UK); cytochrome P450 27A1 (CYP27A1) (SAB1400066) (Sigma-Aldrich, St Louis, MO, USA); YAP (4912S), p-YAP (4911S), LATS1 (9135S), p-LATS1 (9157) (Cell Signaling Technology, Danvers, MA, USA); cyclin A1 (ab65596) (Abcam, Cambridge, UK) and β-Actin (A5316) (Sigma-Aldrich, St. Louis, MO, USA) as a loading control. Signals were detected by enhanced chemiluminescence, using peroxidase-conjugated secondary antibodies and Clarity Western ECL substrate (Bio-Rad Laboratories, Hercules, CA, USA) and quantified with standard densitometric scanning software (Quantity One; BioRad Laboratories, Hercules, CA, USA).

### 2.7. Reverse Transcription and Quantitative Polymerase Chain Reaction

Total RNA was isolated from frozen rat liver and intestine sections using TRIzol reagent (Invitrogen, Madrid, Spain) and was quantified with a NanoDrop 1000 spectrophotometer. Two µg of RNA was reverse transcribed using the high capacity cDNA reverse transcription kit. Real-time PCR was performed in an ABI PRISM 7900 HT detection system by using 5 ul of PowerUp SYBR green master mix (ThermoFisher Scientific, Life Technologies, Carlsbad, CA, USA) in a total of 10 µl amplification mixture, containing 10 ng of reverse-transcribed RNA and 300nM of rat primers. 18S was used as a housekeeping gene. The ΔΔCt method was used for relative quantifications. Data were calculated with respect to the sham group and expressed as percentages. The primer sequences were as follows: *Fgfr4* (forward 5′-CAGTGCTACCCGCAGAGGAAG-3′ and reverse 5′-TGGATTGCTTGTCGGTGA TACA-3′); *Iqgap1* (forward 5′-ACAATCTGGAGACGCAAGCA-3′ and reverse 5′-AGCTGCTCTCGG TTATACGC-3′); *Lats1* (forward 5′-TCTGCATCGCAAGAAGCAAC-3′ and reverse 5′-TCTCATTTG ATCCTGGGCATCT-3′); *Mst1* (forward 5′-GAGACCGTGCAACTGAGGAA-3′ and reverse: 5′-TGA ATCGCCTTGTACACGCT-3′); *Yap* (forward 5′-CCTGATGGATGGGAGCAAGC-3′ and reverse 5′-ACTCTGAGTGATCCTCTGGTTC-3′); *Ctgf* (forward 5′ AGACACATTTGGCCCTGACC-3′ and reverse 5′-TCTTAGAACAGGCGCTCCAC-3′); *18S* (18S ribosomal RNA, forward 5′-GGGAGCCTGAGAAACGGC-3′ and reverse 5′-GGGTCGGGAGTGGGTAATTT-3′).

### 2.8. Liver and Intestine Histology

To assess the severity of hepatic injury, paraffin-embedded liver sections were stained with hematoxylin and eosin, and blind histological scoring was performed by a board-certified pathologist, using a point-counting method on an ordinal scale, as follows: grade 0, minimal or no evidence of injury; grade 1, mild injury consisting of cytoplasmic vacuolation and focal nuclear pyknosis; grade 2, moderate to severe injury with extensive nuclear pyknosis, cytoplasmic hypereosinophilia, and loss of intercellular borders; grade 3, severe necrosis with disintegration of hepatic cords, hemorrhage, and neutrophil infiltration; grade 4, very severe necrosis with disintegration of hepatic cords, hemorrhaging, and neutrophil infiltration [19]. The severity of intestinal damage was scored on a scale from 0 to 5, as described by Chiu et al. [26]. Liver steatosis was visualized by Oil Red staining of liver cryosections. Liver tissues were frozen in optimal cutting temperature (OCT) compounds. The sections were fixed with 10% formalin and the slides were placed in 100% propylene glycol and stained in 0.5% Oil Red solution in propylene glycol. The slides were transferred to an 85% propylene glycol solution and processed for hematoxylin counterstaining. The percentage of steatosis was calculated by image analysis [24]. At least 30 high-power fields were counted per slide. The score described by Brunt et al. [27] is suitable for the assessment of the full spectrum of nonalcoholic fatty liver disease, including simple steatosis. This score is calculated as the weighted sum of the scores for steatosis (the % of liver cells containing fat, range 0–3; 0, <5%; 1, 5–33%; 2, >33–66%; and 3, >66%), ballooning (0–2), lobular inflammation (0–3), and fibrosis state (1, perisinuoidal or periportal; 2, perisunoidal and periportal; 3, briding fibrosis, and 4: cirrhosis). Thus, this score ranges from 0 to 8. The score described by Matteoni et al. [28] is defined as the following types: type 1, fatty liver alone; type 2, fat accumulation and lobular inflammation; type 3, fat accumulation and ballooning degeneration; type 4, fat accumulation, ballooning degeneration, and fibrosis. As previously reported [19,24,29,30], steatosis in Zucker rats used in the current study is not associated with inflammation. Ob rats exhibited moderate macrovesicular and microvesicular fatty infiltration in hepatocytes (40–60% steatosis) but no apparent inflammation or fibrosis. These observations in Ob rats correspond to grade 1 according to Brunt’s classification, and type 1 according to Metteoni’s classification [27,28].

### 2.9. Immunohistochemistry

After fixation with 4% formalin/phosphate-buffered saline, paraffin-embedded livers were sliced and immunostained using mouse monoclonal antibody anti-proliferating cell nuclear antigen (PCNA) (Agilent, Santa Clara, CA, USA) and anti-YAP (Cell Signaling Technology, Danvers, MA, USA). Staining was developed with diaminobenzidine (DAB), using DAKO Envision System (Agilent, Santa Clara, CA, USA) according to the manufacturer’s protocol. Sections were counterstained with hematoxylin. At least 30 high-power fields were counted per slide.

### 2.10. Statistics

Data are expressed as means ± standard error and were statistically analyzed via a one-way analysis of variance, followed by post hoc Student–Newman–Keuls test. *p* < 0.05 was considered significant.

## 3. Results

### 3.1. Role of FGF15 in Recipients of Non-Steatotic and Steatotic Liver Grafts from DCDs

**Figure 1:**
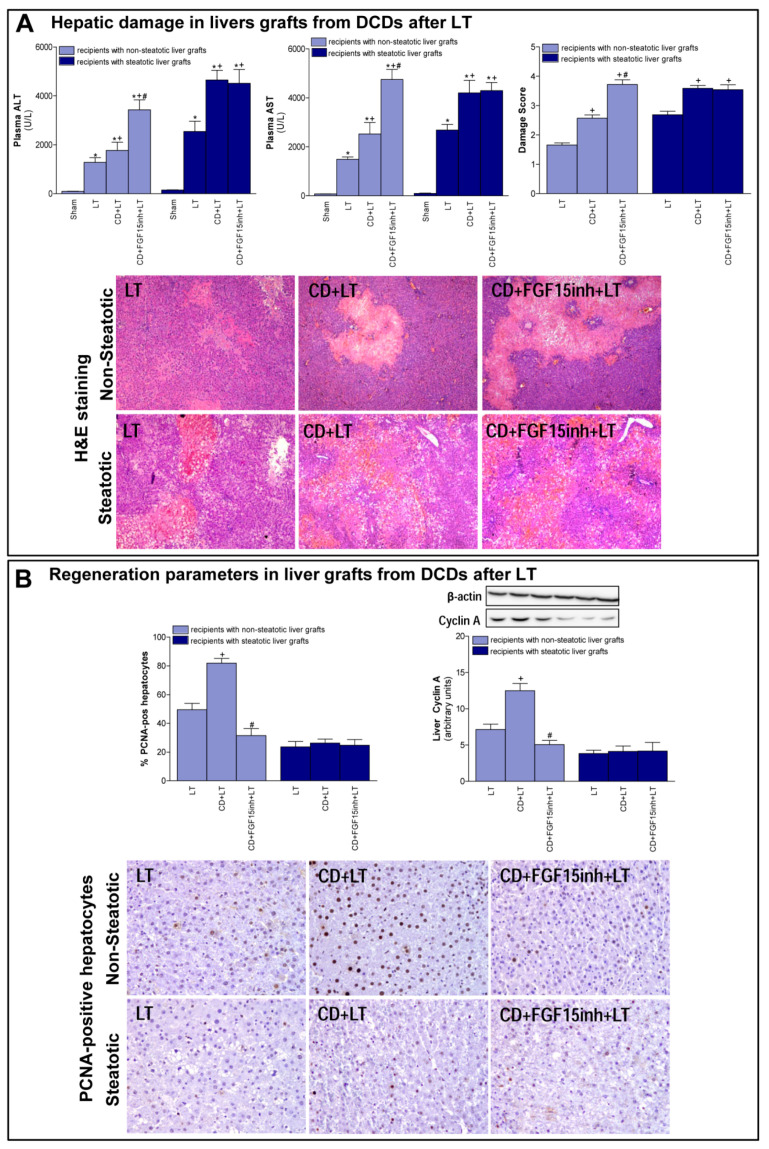
Liver injury and regeneration in non-steatotic and steatotic livers from DCDs after LT. (**A**) Hepatic damage parameters: plasma transaminases, liver damage score and representative photographs of histological changes in LT from DCDs. In non-steatotic grafts, LT exhibited moderate and multifocal areas of coagulative necrosis, the extent and the number of necrotic areas increased in CD + LT, and extensive and confluent areas of coagulative necrosis were observed in CD + FGF15inh + LT. In steatotic grafts, LT showed extensive and confluent areas of coagulative necrosis, whereas such coagulative necrosis was even more severe in CD + LT and CD + FGF15inh + LT (4×). (**B**) Liver regeneration parameters: percentage of positive hepatocytes of PCNA, protein expression of cyclin A1 in liver tissue, and representative photographs of immunohistochemical staining of PCNA-positive cells. In non-steatotic livers, CD + LT increased the number of PCNA-positive hepatocytes compared with the LT group. CD + FGF15inh + LT showed fewer positive cells than the CD + LT group. In steatotic livers, PCNA-positive cells were similar in LT, CD + LT and CD + FGF15inh + LT groups (20×). * *p* < 0.05 vs. Sham; + *p* < 0.05 vs. LT; # *p* < 0.05 vs. CD + LT.

First, we determined the effect of CD on damage and regeneration in both non-steatotic and steatotic liver grafts after transplantation. CD was seen to exacerbate damage in both types of grafts since transaminase levels and damage score values were higher in the CD+LT group than in the LT group (LT without CD) (Figure 1A). The histological study of liver grafts from DCDs (CD + LT) showed moderate and multifocal areas of coagulative necrosis and neutrophil infiltration, randomly distributed through the parenchyma, in non-steatotic livers whereas in steatotic grafts, severe, extensive, and confluent areas of coagulative necrosis were observed (Figure 1A). In LT using non-steatotic grafts, CD triggered an increment in regeneration markers, PCNA and cyclin A (CD + LT group), compared to those without CD (LT group). On the other hand, in steatotic livers undergoing transplantation, regeneration parameters were unchanged by CD (Figure 1B).

**Figure 2:**
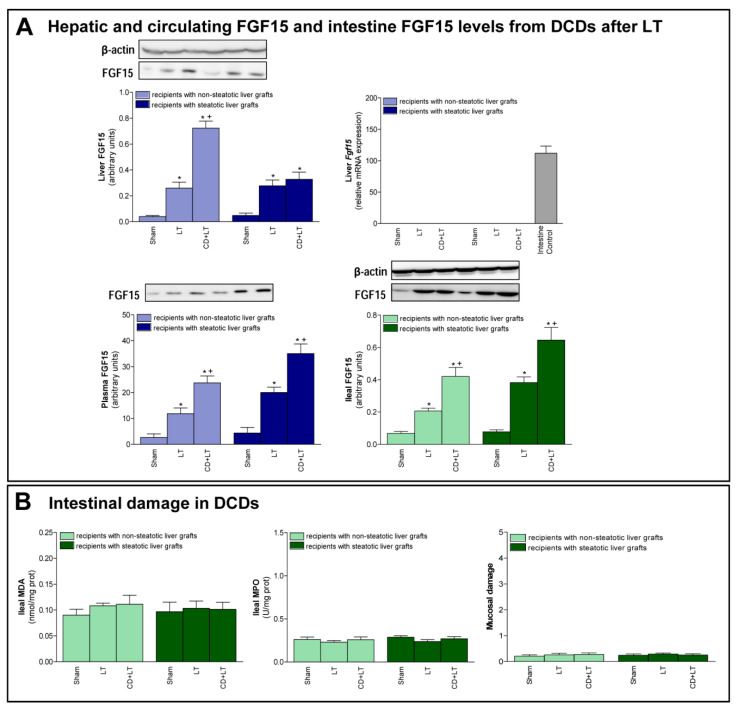
FGF15 levels and intestinal damage in LT with DCDs. (**A**) FGF15 protein expression in liver, intestine, and plasma. (**B**) Intestinal injury parameters: MDA, MPO and histological injury according to Chiu-score. * *p* < 0.05 vs. Sham; + *P* < 0.05 vs. LT.

Second, we evaluated the role of FGF15 in damage and regeneration in grafts from DCDs after transplantation. In non-steatotic liver grafts from DCDs, our findings indicated a rise in FGF15 protein levels, in comparison to the results of the LT group. On the other hand, in steatotic livers, FGF15 protein levels were similar in both the CD + LT and LT groups (Figure 2A). Circulatory FGF15/19 is assumed to come from the intestine, but, under conditions of obstructed bile flow, FGF15/19 is also synthesized by the liver [31,32]. However, in our experiment, *Fgf15* was not expressed by the liver in any of the study groups, which was confirmed by a positive control (intestine from the Sham group) (Figure 2A).

When we inhibited the action of FGF15, the non-steatotic grafts from DCDs showed exacerbated damage and impaired regenerative response. Indeed, (a) increased levels of transaminases and damage score values, and (b) reduced PCNA and cyclin A, were observed in the CD + FGF15inh + LT group when compared to the CD + LT group. However, the inhibition of FGF15 action in steatotic livers from DCDs did not induce changes in either hepatic damage or regeneration. Thus, in such types of liver graft transaminases, damage scores, PCNA and cyclin A values were similar in both the CD + FGF15inh + LT and CD + LT groups (Figure 1).

Control experiments based on the administration of FGF15inh in LT without CD were also performed. The inhibition of FGF15 action in LT (FGF15inh + LT group) resulted in parameters of proliferation and damage in non-steatotic livers similar to those of the LT group. For instance, the results corresponding to transaminases and PCNA were the following: ALT levels: 1345 ± 201 and 1281 ± 191 U/L for the FGF15inh + LT and LT groups, respectively; AST levels: 1547 ± 101 and 1488 ± 97 U/L for the FGF15inh + LT and LT groups, respectively; and PCNA: 52.47 ± 4.68 and 49.5 ± 4.42% PCNA-positive hepatocytes for FGF15inh + LT and LT groups, respectively; *p* > 0.05 for all determinations, data not shown in graphs. Similarly, the inhibition of FGF15 action in LT (FGF15inh + LT group) resulted in parameters of proliferation and damage in steatotic livers similar to those of the LT group. For instance, the results corresponding to transaminases and PCNA were the following: ALT levels: 2671 ± 441 and 2544 ± 420 U/L for the FGF15inh + LT and LT groups, respectively; AST levels: 2790 ± 244 and 2683 ± 235 U/L for the FGF15inh + LT and LT groups, respectively; and PCNA: 25.05 ± 3.97 and 23.63 ± 3.74% PCNA-positive hepatocytes for the FGF15inh + LT and LT groups, respectively; *p* > 0.05 for all determinations, data not shown in graphs.

### 3.2. FGF15 Levels in Intestine and Plasma in LT from DCDs

Next, we evaluated the possible contribution of intestine in FGF15 signaling in LT from DCDs. Similar intestinal tissue histology and levels of MDA, MPO were found in the Sham, LT, and CD + LT groups, suggesting that there is no intestinal damage when LT is performed nor when CD is induced, in any type of liver (Figure 2B). Regarding FGF15, an intestinal rise in protein levels was observed in both non-steatotic and steatotic grafts in the CD + LT group when compared to levels recorded in the LT group. This pattern of levels of FGF15 in intestinal tissue was mirrored in circulatory FGF15: levels of FGF15 in plasma were higher in the CD + LT group compared to the LT group, in both non-steatotic and steatotic grafts (Figure 2A).

### 3.3. The Effects of FGF15 on the BA Synthesis Pathway in LT from DCDs

**Figure 3:**
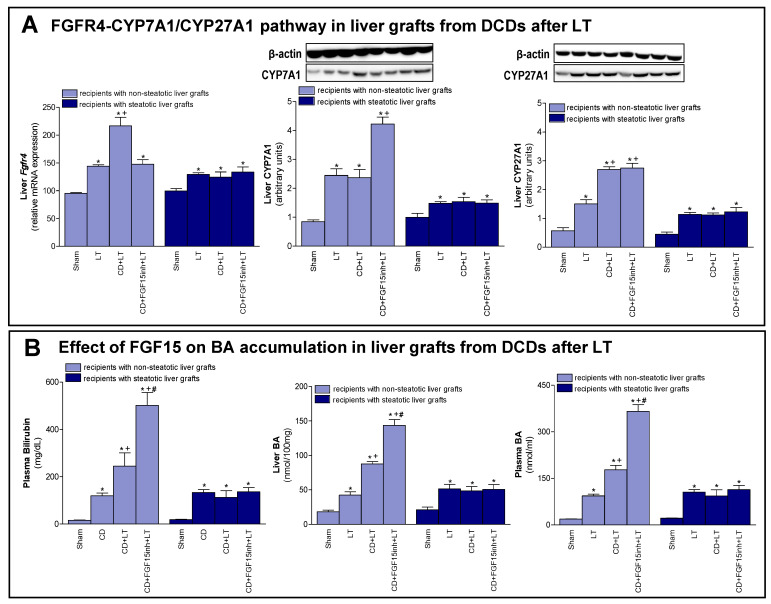
Effect of FGF15 on FGFR4-CYP7A1/CYP27A1 pathway and BA accumulation in LT with DCDs. (**A**) mRNA expression of *Fgfr4* and protein expression of CYP7A1 and CYP27A1 in liver. (**B**) Bilirubin in plasma, and BAs in liver and plasma. * *p* < 0.05 vs. Sham; + *p* < 0.05 vs. LT; and # *p* < 0.05 vs. CD + LT.

As previously mentioned, FGF15/19 regulates BA synthesis through FGFR4 signaling [7,9]. We registered an increased expression of *Fgfr4* in non-steatotic livers when comparing the CD + LT and LT groups. In steatotic grafts, these groups showed no difference in gene expression of *Fgfr4* (Figure 3A). Interestingly, bilirubin and both hepatic and plasma BAs were also higher only in non-steatotic LT from DCDs (Figure 3B). In fact, in the presence of steatosis, such parameters in the CD + LT group resembled the levels found in the LT group. To investigate the signaling pathway associated with the increase in the BA pool observed in non-steatotic LT from DCDs, the classic and alternative BA synthesis pathways were evaluated. The protein expression of CYP7A1, the main enzyme of the classic BA synthesis pathway, was similar in non-steatotic grafts in the CD + LT and LT groups (Figure 3A). On the other hand, protein expression of CYP27A1, a representative enzyme of the alternative BA synthesis pathway, was higher in non-steatotic liver grafts in the CD + LT group than the LT group. In steatotic liver grafts from CD + LT group, CYP7A1 and CYP27A1 protein expression were no different from the LT group (Figure 3A).

Then, we attempted to clarify the possible implications of FGF15 for the synthesis pathways of BAs. As previously demonstrated, when we inhibited the action of FGF15 (CD + FGF15inh + LT), hepatic damage in non-steatotic grafts from DCDs was exacerbated (Figure 1). In the same type of liver, blocking the action of FGF15 (CD + FGF15inh + LT group) reduced the expression of *Fgfr4* and resulted in high levels of bilirubin and hepatic and plasma BAs compared to the CD + LT group. This was related to an overexpression of CYP7A1 in the CD + FGF15inh + LT group compared to the CD + LT group, whereas CYP27A1 did not alter its expression (Figure 3). As we saw for damage, the inhibition of FGF15 action in steatotic LT from DCDs (CD + FGF15inh + LT) did not induce changes in either *Fgfr4*, bilirubin, BAs, CYP7A1 or CYP27A1 levels when compared to results from the CD + LT group.

### 3.4. The Effect of FGF15 on the Hippo/YAP Signaling Pathway in Liver Grafts from DCDs after LT

**Figure 4:**
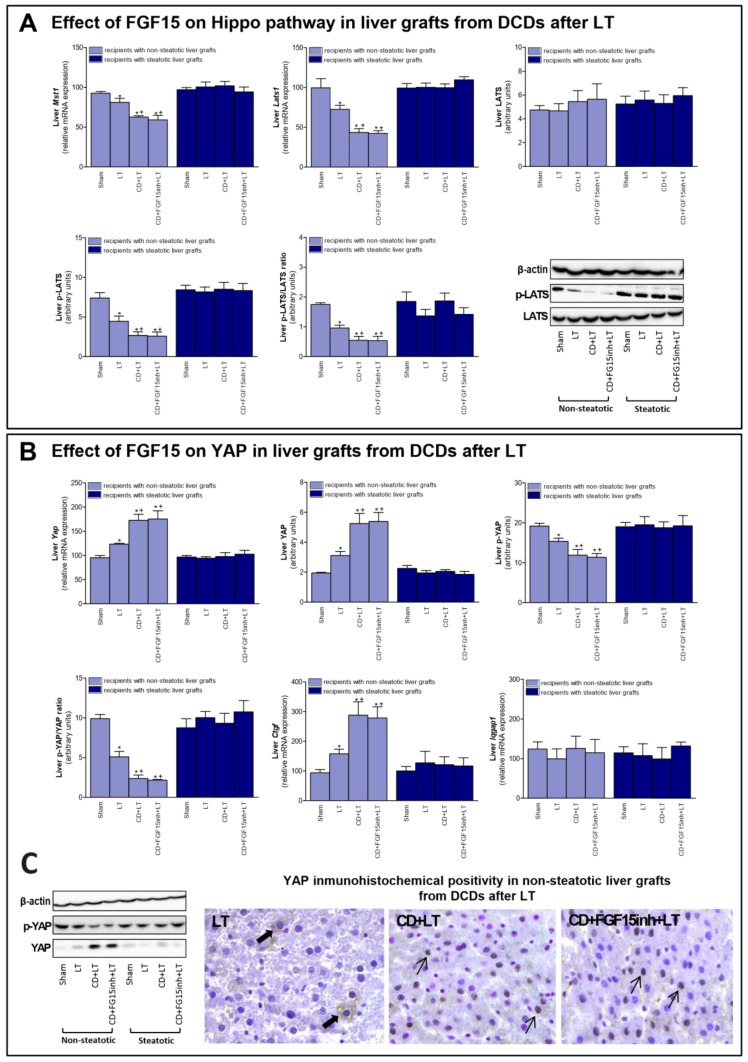
Effect of FGF15 on Hippo/YAP signaling pathway in LT with DCDs. (**A**) mRNA expression of *Mst1*, *Lats1*, and protein levels of LATS, p-LATS, and p-LATS/LATS ratio in liver. (**B**) mRNA levels of *Yap,* protein levels of YAP, p-YAP, p-YAP/YAP ratio; mRNA expression levels of *Ctgf and Iqgap1* in liver. * *p* < 0.05 vs. Sham and + *p* < 0.05 vs. LT; # *p* < 0.05 vs. CD + LT. (**C**) Representative photomicrographs of YAP immunohistochemical positivity. Inmunostaining of cytoplasmatic YAP in hepatocyte (thick arrow) of LT group and YAP nuclear staining in CD + LT and CD + FGF15Inh + LT groups (thin arrow) (40×).

FGF15/19 regulates liver regeneration through Hippo/YAP signaling [11]. Our results indicated that mRNA expression of mediators of Hippo signaling such as *Mst1 and Lats1*; and protein levels of the final effector LATS (in its phosphorylated form), were decreased in non-steatotic grafts in the CD + LT group, when compared to the LT group (Figure 4A). In contrast, mRNA expression and protein levels of YAP were higher, and p-YAP protein levels lower, in the CD + LT group (Figure 4B). Furthermore, YAP expression was markedly higher in hepatocyte nuclei in the CD + LT group. This coincided with over-expression of YAP target genes, such as connective tissue growth factor (*Ctgf*). In non-steatotic liver grafts, the mRNA expression of *Mst1*, *Lats1,* and *Ctgf* and the protein expression of p-LATS and p-YAP, and the YAP immunohistochemical positivity were similar, in the CD + FGF15inh + LT and CD + LT groups. In steatotic grafts, the expression of all the parameters of the Hippo/YAP signaling pathway was unchanged across all experimental groups (Figure 4). BA levels, through the IQ motif containing GTPase activating protein 1 (IQGAP1), are able to induce YAP activation [33,34]. However, in our hands, *Iqgap1* mRNA expression was similar across all groups of the study, for both liver types (Figure 4B).

### 3.5. FGF15 and Related Signaling Pathways in Liver Grafts from DCDs before Procurement

**Figure 5:**
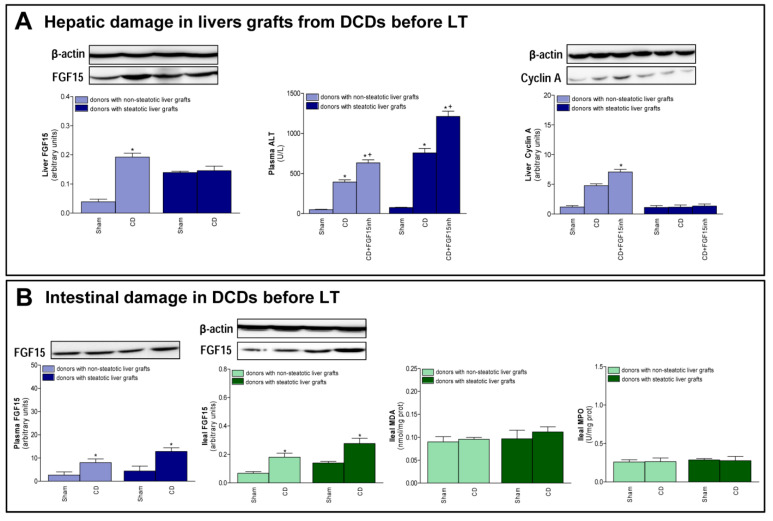
FGF15 levels and intestinal damage before procurement from DCDs. (**A**) FGF15 levels in liver and liver injury and regeneration parameters. (**B**) FGF15 in plasma and intestine and MDA and MPO in intestine. * *p* < 0.05 vs. Sham; + *p* < 0.05 vs. CD.

The changes recorded after transplantation, with respect to FGF15 and associated signaling pathways, were also observed immediately after the induction of CD, just before the procurement of grafts. In non-steatotic livers, a rise in FGF15 levels was observed in the CD group, when compared to the Sham group (Figure 5A). On the other hand, in steatotic livers, there was no difference in FGF15 levels between those experimental groups. Induction of CD increased transaminases in both non-steatotic and steatotic livers from the CD group compared to the Sham group. Also, we observed an increment in cyclin A protein expression in the CD group with non-steatotic livers, but this regeneration parameter was unchanged in groups of steatotic grafts (Figure 5A). Increased transaminase levels and reduced cyclin protein expression were recorded in the CD + FGF15inh group, when compared to CD group, in non-steatotic livers. In steatotic livers, there were no differences in either hepatic injury or regeneration parameters between the CD + FGF15inh and CD groups (Figure 5A). A rise in FGF15 intestinal levels was observed in the CD group when compared to the Sham group and this was mirrored in circulatory FGF15 (Figure 5B). Induction of CD in donors did not lead to intestinal injury prior to the procurement of liver grafts.

## 4. Discussion

The current study shows that CD exacerbates damage in both non-steatotic and steatotic LT. After hepatic damage, caused by liver disease, trauma, or partial hepatectomy, an increased proliferative response is induced in order to repair the damaged hepatic tissue [35]. This was found to occur in non-steatotic livers from DCDs since damage was associated with an increased proliferative response when compared to non-DCDs. The signaling events that characterize the hepatic regeneration culminate in cyclin-dependent hepatocyte re-entry into, and progression through, the cell cycle. As part of this process, hepatocellular cyclin A mediates the G1/S transition [36]. In non-steatotic grafts from DCDs, cyclin A and PCNA-positive staining cells were increased. This was not the case with steatotic livers because, in spite of the exacerbated hepatic damage induced by CD, the presence of steatosis prevented the capacity of the liver to induce a proliferative response aimed at reparing damage.

The current study showed evidence that increased FGF15 levels in intestine were triggered by CD and released from the ileum into the bloodstream. Nevertheless, the increased circulatory FGF15 induced by CD was reflected in increased hepatic FGF15 only in non-steatotic livers. The current study provides evidence of the importance in enterohepatic pathway regulation to maintain high FGF15 in non-steatotic livers from DCDs, to protect against damage and regenerative failure. Indeed, the inhibition of FGF15 action exacerbated hepatic damage and proliferative response failure in non-steatotic LT from DCDs. However, the FGF15 signaling pathway does not seem to be crucial to protect against damage and regenerative failure in the presence of steatosis. Indeed, under CD conditions, intestine-derived FGF15 reached the circulation but was not accumulated in steatotic grafts. In addition, when FGF15 action was inhibited, no changes were observed in either damage or proliferative response parameters in steatotic grafts. In our view, an increase in the release of FGF15 from the steatotic liver to the circulation as well as the blocking of the uptake of FGF15 by the steatotic liver from the circulation might explain, at least partially, the disparity between hepatic and circulatory FGF15 levels in steatotic LT from DCDs. The capacity of the liver to clear FGF15 remains to be clarified [37].

It has been reported that lack of FGF15 (*Fgf15−/−* mice) results in increased hepatic steatosis and the development of endoplasmic reticulum stress in the liver of mice fed a high fat diet; and in the same study, Alvarez-Sola et al. demonstrated how Fibapo treatment (a chimeric molecule based on the fusion of FGF19 with apolipoprotein A-I) protects from lipid-mediated cellular stress and damage in obese db/db mice undergoing partial hepatectomy without I/R [38]. There is another study also demonstrating profound antisteatotic, anti-inflammatory, and antifibrotic activities of a therapy based on the administration of engineered FGF19 variant in diet-induced mouse models of non-alcoholic steatohepatitis (NASH) [39]. In contrast to these studies, here we describe for the first time that FGF15 does not play a role in injury observed steatotic livers undergoing CD and LT in Ob Zucker rats. This differential role of FGF15 is not surprising, considering the different animal species and experimental models of steatosis: mice with hepatic steatosis induced by diet and obese db/db mice (aggressive model of type 2 diabetes and obese phenotype) in the studies reported by Alvarez-Sola et al. and Zhou et al. [38,39] versus genetically Ob Zucker rats in our study. We used Ob Zucker rats since induced genetic models of steatosis with these rats are not associated with parameters of inflammation [40,41]. Thus, in our experimental conditions, this induced genetic model of steatosis is an appropriate experimental model for investigating the role of FGF15 in hepatic damage, inflammation, and regenerative failure associated with LT from DCDs. On the other hand, in other experimental models of steatosis induced by diet, the steatosis is associated with inflammation [39,42] and/or with diabetes [38], which may conceal the inflammation and other deleterious effects associated with hepatic I/R by itself. It should also be noted the differences in the db/db mice used by Alvarez-Sola et al. versus experimental genetically Ob Zucker rats used in our study. All these observations on the experimental models of steatosis should be considered since the molecular signaling involved in liver surgery depend on the type of steatosis [40,41]. In addition, steatotic mice used by Zhou et al. were not submitted to any surgical procedure [39], Alvarez-Sola et al. evaluated steatotic livers subjected to partial hepatectomy without I/R [38] and we studied steatotic livers undergoing LT from DCDs. This should be considered since important differences in the response to one pharmacological treatment in steatotic livers depending on the type of surgical procedure have been described. This is, for example, the case for the effects of visfatin, resistin, and angiotensin II in steatotic liver submitted either to hepatic resections or LT [43,44,45,46]. Undoubtedly, CD and I/R associated with LT are two pathological conditions that involve complex signaling networks, and when combined, they interact and might result in new signaling pathways that are not described until date. Moreover, it should be taken into account that to evaluate the role of endogenous FGF15, we inhibited its action whereas Alvarez-Sola et al. used *Fgf15−/−* mice or an exogenous engineered FGF19 variant, Fibapo. It is important to mention that the chimeric molecule, Fibapo, used by Alvarez-Sola et al. [38] has been modified to enhance the half-life. Indeed, the serum half-life of Fibapo was 8.5 h, significantly higher than that of FGF15/19 (1.8 h). Zhou et al. used other FGF19 analogue called M70 that differs from wild-type FGF19 in the amino terminus, a key region of the protein involved in receptor interactions and signaling modulation [39]. Then, the possibility of differential effects of endogenous FGF15 (as we evaluated in the present study) and exogenous Fibapo and M70 (used by Alvarez-Sola and Zhou, respectively) should not be discarded. Thus, taking into account all these observations, it is not surprising that the results reported in this study about FGF15/19 do not follow the same pattern as those described in the literature for other kinds of animals, experimental models of steatosis, and surgical conditions that those described in the current study.

The differential effects resulting from the FGF15 action inhibition between LT with or without CD are expected and of scientific and clinical interest. Amongst other variables, living, brain dead, and CD donors include different profound physiological and structural derangements. Consequently, specific pathological mechanisms and protective strategies might be required in LT depending on the characteristic of liver grafts (steatotic versus non-steatotic) and type of donor (living, brain death, or CD donors). Thus, the use of experimental models of LT from DCDs, as we have done in the current study, is required if our aim is to evaluate the role of FGF15 in the deleterious effects of CD in both steatotic and non-steatotic LT. In our view, the use of appropriate experimental models of LT is relevant to elucidate the pathological mechanisms involved in hepatic I/R injury and consequently for the establishment of useful strategies in clinical LT. It should be noted that a relatively low percentage of organs are acquired from living donors (without CD or brain death) in clinical practice.

The maintenance of BA concentration is needed to avoid toxicity as BAs are also detergents that may damage cellular membranes and trigger hepatocellular death [35]. Accumulation of BAs in the liver is toxic and can lead to bile duct epithelium damage, hepatocyte death, inflammation, and liver dysfunction. Thus, as previously reported, strategies aimed at reducing hepatic BAs are key in preventing liver damage and slowing disease progression [39]. Of interest, non-steatotic grafts from DCDs showed higher bilirubin and BA levels than non-DCDs. This was associated with the accumulation of hepatic FGF15 from the intestine. In our view, the accumulation of FGF15 in non-steatotic grafts acts as a compensatory response to try to protect the liver against CD by BA suppression through FGFR4. Indeed, if FGF15 action is inhibited, BAs are accumulated in non-steatotic grafts from DCDs resulting in exacerbated hepatic damage. Our results are in accordance with previous experimental and clinical reports showing that under damage conditions, such as cholestasis [32] and hepatectomy [47], significant amounts of endogenous hepatic FGF15/19 provide a protective adaption against BA overload. It is known that high BA levels are signals to stimulate the intestinal generation of FGF15 to protect against the deleterious effects of BA [48]. According to our results, CD did not induce alterations in either bilirubin or BA levels in steatotic livers. Thus, as expected, an accumulation of hepatic FGF15 in the presence of steatosis is not required to protect against the deleterious effects of the BA pool. On the other hand, we observed that in steatotic LT from DCDs, the intestine generated FGF15 despite low hepatic BA levels and the minor role of FGF15 in hepatic damage and regeneration. Thus, in our view, the increased levels of circulatory FGF15 (derived from the intestine) in steatotic LT from DCDs may be required for the exertion of its action in target organs other than the liver, such as adipose tissue. Indeed, adipose tissue shows receptors for FGF15. It should be noted that obesity induces functional changes in adipose tissue, and the presence of hepatic steatosis alters its uptake of different mediators from the circulation [49]. Further investigation will be required to elucidate this.

FGF15/19 is an important regulator of BA synthesis through its regulation of CYP7A1, which is the rate-limiting enzyme for BA synthesis [48]. In addition to this classic BA synthetic pathway initiated by CYP7A1, the alternative pathway synthesis of BAs initiated by CYP27A1 should be considered [9]. Indeed, in liver diseases such as cirrhosis, the alternative pathway can become predominant [50,51]. In our hands, when compared to the results of non-steatotic LT from non-DCDs, the high BA levels observed might be explained by the high protein expression of CYP27A1 rather by changes in CYP7A1. In addition, in non-steatotic LT from DCDs, endogenous FGF15 seemed to exert its action only by regulating CYP7A1. Indeed, inhibiting FGF15 action caused an increment in CYP7A1, which triggered the increment in BA accumulation, whereas it did not induce changes in CYP27A. The effects of endogenous FGF15 on CYP7A1 regulation might contribute partially to reducing the BA accumulation in non-steatotic grafts under CD conditions. Indeed, although we observed increased levels of FGF15 in non-steatotic liver from DCDs, CYP7A1 was still overexpressed. Moreover, FGF15 was unable to regulate the alternative pathway of BA synthesis involving CYP27A1. All of this resulted in BA accumulation and damage in non-steatotic LT from DCDs.

Since BA are liver-specific metabolites, they have been considered potential markers of liver injury in several metabolomics studies [52,53], and in fact it has been suggested that BA are inflammatory mediators at pathological concentrations [54]. Reports in the literature have shown that BAs act as inflammagens and directly activate signaling pathways in hepatocytes that regulate the production of pro-inflammatory mediators that stimulate the recruitment of neutrophils into the liver. Exposure of hepatocytes to BAs increased levels of numerous mediators, including cytokines as interleukin (IL)-1β, IL-10, chemokines as keratinocyte chemoattractant (KC), interferon gamma-induced protein 10 (IP-10), interferon-inducible T-cell alpha chemoattractant (I-TAC), monocyte chemoattractant protein (MCP)-1, regulated upon activation, normal T cell expressed and presumably secreted (RANTES), MCP-3, macrophage inflammatory protein (MIP)-1α, MIP-2, MIP-3α, LPS-induced CXC chemokine (LIX), scavenger receptor for phosphatidylserine and oxidized low-density lipoprotein (SR-PSOX), B lymphocyte chemoattractant (BLC), adhesion molecules as intercellular adhesion molecule-1 (ICAM-1), vascular cell adhesion molecule-1 (VCAM-1), enzymes in arachidonic acid metabolism (cyclooxygenase-2 [COX-2]), and other proteins that influence immune cell levels and function as plasminogen activator inhibitor-1 (PAI-1), urokinase plasminogen activator receptor (uPAR), granulocyte colony-stimulating factor (G-CSF), and granulocyte macrophage colony-stimulating factor (GM-CSF) [54]. Since several of such mediators are widely known to contribute to hepatic damage in several models of I/R with non-steatotic livers [55,56], it is reasonable to conceive that accumulation of BAs directly activate proinflammatory signaling networks that finally lead to severe liver injury in non-steatotic livers undergoing CD and LT. In addition, BAs have been shown to exert its effects on a multitude of signaling pathways in hepatocytes. For example, p38, c-Jun N-terminal kinase are activated in BA-treated hepatocytes and livers of BDL mice, and BAs also can activate various isoforms of the protein kinase C (PKC) family and pregnane X receptor [57,58,59,60,61,62]. Given that, a relevant role for mitogen-activated protein kinases and PKC in hepatic I/R injury is well established [60,63,64], we hypothesize that BAs promote pro-inflammatory mediators through one or more of these signaling pathways in non-steatotic LT from DCDs.

The next step was to investigate the underlying mechanisms by which endogenous FGF15 promotes regeneration in non-steatotic LT from DCDs. The Hippo pathway inhibits a proliferative response since it prevents the nuclear accumulation of YAP and activation of proliferation promoting genes [11]. YAP has been identified as the master regulator of hepatic cell proliferation [65]. In our hands, we observed a reduction in pLATS and the p-LATS/LATS ratio in non-steatotic LT from DCDs. This was associated with an increase in YAP activity, since p-YAP and the pYAP/YAP ratio were decreased, resulting in increasing nuclear localization of YAP. The expression of targets downstream of YAP, such as *Ctgf* was also increased. Thus, CD may induce YAP activity to promote the reparation process, counteracting the damage induced by CD in non-steatotic liver grafts. However, when FGF15 action was inhibited, the proliferative response was impaired but that did not induce changes in the Hippo/YAP pathway. This indicates that the benefits of endogenous FGF15 on the proliferative response is not dependent on changes in the Hippo/YAP pathway. Studies of different liver diseases, including tumorigenesis [13,34,66], indicate that elevated BA levels induce YAP activation through the IQGAP1. Our results seem to indicate the absence of a potential relationship between BA accumulation and YAP activation in the effects of FGF15 in non-steatotic LT from DCDs. Indeed, in non-steatotic LT with inhibition of FGF15 action, we observed increases in the levels of BAs but no changes in YAP activity. In addition, IQGAP1 expression was similar across all groups of the study, independent of BA levels. Thus, in contrast to studies of pathologies that were quite different from those reported herein, including tumorigenesis [11,33,34], the BA–IQGAP1–YAP signaling pathway is not involved in the benefits of endogenous FGF15 in non-steatotic LT from DCDs.

The extracellular signal-regulated protein kinase (ERK1/2) pathway is one of the most precise pathways by which FGFR4, the receptor of FGF15/19, mediates the proliferative signal from the cytomembrane into the nucleus [67]. In fact, FGF15/19 has demonstrated to promote hepatocyte proliferation in vitro and enhanced the expression of cyclin D1, cyclin A, and cyclin E, and such effect occurred through the FGFR4/ERK1/2 pathway. This signaling mechanism could be involved in the effects of FGF15/19 on cell proliferation in the experimental conditions evaluated herein. As demonstrated, lack of FGF15/19 resulted in decreased FGFR4 and cyclin A in non-steatotic livers undergoing CD and I/R injury associated with LT. The important role for ERK1/2 in LT and experimental models of liver regeneration in non-steatotic livers has been previously demonstrated [45,68,69,70,71,72,73]. Thus, it could be bearing in mind the possibility that failure in cell proliferation induced by FGF15 inhibition could be related to a reduced activation of ERK1/2.

**Figure 6:**
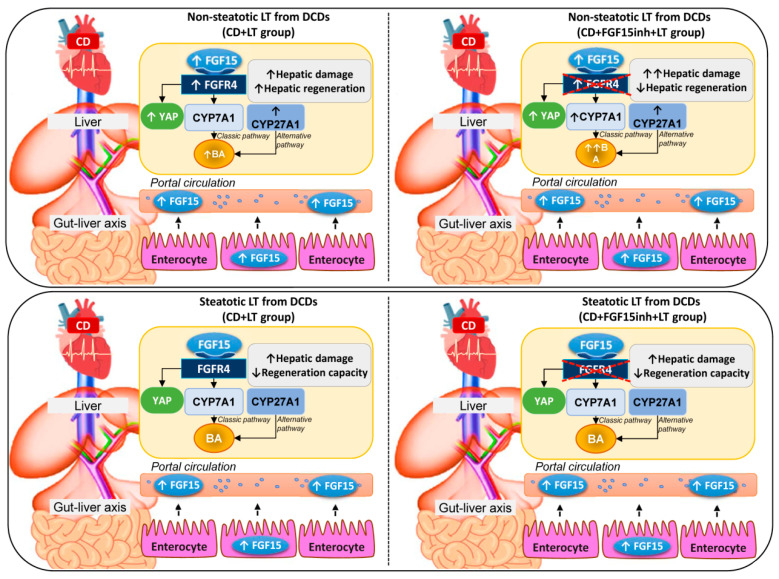
Schematic representation showing the effect of the different interventions, depicting outcomes and proposed signaling pathways of the current study. The results of CD + LT were compared with those of the LT group. The results of CD + FGF15inh + LT were compared to those of the CD + LT group.

In light of the above-mentioned results, in non-steatotic LT from DCDs, increased intestinal and circulatory FGF15 was associated with increased hepatic FGF15. FGFR4 was up-regulated, whereas CYP7A1 and CYP27A1 were overexpressed, resulting in hepatic and circulatory BA accumulation (Figure 6). Moreover, in non-steatotic LT, CD activated the regenerative pathway, Hippo/YAP. This resulted in damage and an increased regenerative response in liver grafts from DCDs. When FGF15 action was inhibited, it triggered the enzymatic machinery based on CYP7A1 up-regulation, whereas CYP27A1 expression was unchanged. This exacerbated BA accumulation and hepatic damage. Moreover, blocking FGF15 action led to impairment through regenerative failure in non-steatotic LT from DCDs, whereas the Hippo/YAP pathway was unaltered. Consequently, in non-steatotic LT from DCDs, endogenous FGF15 protects against damage and promotes proliferation. FGF15 acts only through the enzyme CYP7A1, involved in the classical BA synthesis pathway, whereas the enzyme CYP27A1, involved in the alternative pathway triggered by CD, would still work, causing high levels of BAs, and thus inducing damage in non-steatotic grafts from DCDs. Moreover, FGF15 promotes liver regeneration by a mechanism that is independent of the Hippo/YAP signaling pathway. In steatotic LT, CD was associated with increased intestinal and circulatory FGF15 levels while FGF15 was not accumulated in liver grafts. This was associated with either with unchanging levels of FGFR4, CYP7A1, CYP27A1, and hepatic and circulating BA, or with the lack of alteration in the Hippo/YAP pathway. The result was exacerbated damage and a lack of proliferative capacity in steatotic grafts from DCDs. When FGF15 action was inhibited, FGFR4, CYP7A1, CYP27A1, hepatic and circulating BA, and the Hippo/YAP pathway were unaltered; no effects on either hepatic damage or regenerative response were evidenced in steatotic LT from DCDs. In consequence, in steatotic LT from DCDs, endogenous FGF15 plays a minor role in the vulnerability of the liver to damage and regenerative failure. From the point of view of clinical application, therapeutic intervention in LT from DCDs based on endogenous FGF15 action regulation might be useful for non-steatotic liver grafts but not in the presence of steatosis.

## Abbreviations

ALT	Alanine aminotransferase
AST	Aspartate aminotransferase
BAs	Bile acids
BLC	B lymphocyte chemoattractant
CD	Cardiocirculatory death
COX-2	Cyclooxygenase 2
*Ctgf*	Connective tissue growth factor
CYP7A1	Cytochrome P450 7A1
CYP27A1	Cytochrome P450 27A1
DAB	Diaminobenzidine
DCDs	Donors after cardiacirculatory death
ERK1/2	Extracellular signal-regulated protein kinase
FGF15	Fibroblast growth factor 15
FGF19	Fibroblast growth factor 19
FGFR4	Fibroblast growth factor receptor 4
G-CSF	Granulocyte colony stimulating factor
GM-CSF	Granulocyte macrophage colony stimulating factor
I/R	Ischemia-reperfusion
ICAM-1	Intercellular adhesion molecule-1
IP-10	Interferon gamma-induced protein 10
IQGAP1	IQ motif containing GTPase activating protein 1
I-TAC	Interferon-inducible T-cell alpha chemoattractant
KC	Chemokines as keratinocyte chemoattractant
LATS1/2	Large tumor suppressor 1 and 2
LIX	LPS-induced CXC chemokine
Ln	Lean
LT	Liver transplantation
MCP	Monocyte chemoattractant protein
MIP	Macrophage inflammatory protein
MDA	Malondialdehyde
MPO	Myeloperoxidase
MST1/2	Mammalian sterile-20 kinase 1 and 2
NASH	Non-alcoholic steatohepatitis
Ob	Obese
OCT	Optimal cutting temperature
PAI-1	Plasminogen activator inhibitor-1
PCNA	Proliferating cell nuclear antigen
PKC	Protein kinase C
RANTES	Regulated upon activation, normal T cell expressed and presumably secreted
SR-PSOX	Scavenger receptor for phosphatidylserine and oxidized low density lipoprotein
uPAR	Urokinase plasminogen activator receptor
UW	University of wisconsin
VCAM-1	Vascular cell adhesion molecule-1
YAP	Yes-associated protein
