# Correction: Avalos-de León et al. The Effect of Fibroblast Growth Factor 15 Signaling in Non-Steatotic and Steatotic Liver Transplantation from Cardiocirculatory Death. *Cells* 2019, *8*, 1640

**DOI:** 10.3390/cells13131139

**Published:** 2024-07-03

**Authors:** Cindy G. Avalos-de León, Mónica B. Jiménez-Castro, María Eugenia Cornide-Petronio, José Gulfo, Floriana Rotondo, Jordi Gracia-Sancho, Araní Casillas-Ramírez, Carmen Peralta

**Affiliations:** 1 Institut d’Investigacions Biomèdiques August Pi I Sunyer (IDIBAPS), 08036 Barcelona, Spain; avalosdl.cin@gmail.com (C.G.A.-d.L.); monicabjimenez@hotmail.com (M.B.J.-C.); cornide@clinic.cat (M.E.C.-P.); gulfo@clinic.cat (J.G.); floriana.rotondo@gmail.com (F.R.); 2 Centro de Investigación Biomédica en Red de Enfermedades Hepáticas y Digestivas (CIBERehd), 08036 Barcelona, Spain; jordi.gracia@idibaps.org; 3 Liver Vascular Biology Research Group, IDIBAPS, 08036 Barcelona, Spain; 4 Hospital Regional de Alta Especialidad de Ciudad Victoria “Bicentenario 2010”, Ciudad Victoria 87087, Mexico; 5 Facultad de Medicina e Ingeniería en Sistemas Computacionales de Matamoros, Universidad Autónoma de Tamaulipas, Matamoros 87300, Mexico

In the original publication [1], an error was detected in the Western blot image corresponding to the β-actin used as a loading control for CYP27A1 (Figure 3A). Due to human error, when preparing the figures of our manuscript, the Western blot image of the LATS protein (Figure 4A) was accidentally also included as the image corresponding to the β-actin used as a loading control for the CYP27A1 protein.

Despite this error, the image does not change the results or conclusions of the manuscript at all, since the densitometric quantification of CYP27A expression (which was shown as a bar graph below the representative Western blot images in the published version of the manuscript) was performed including the densitometric values of the correct β-actin used as a loading control for CYP27A. In the present correction of the manuscript, the correct representative Western blot image of the β-actin used as a CYP27A1 loading control is included in Figure 3A.

It is important to mention that, in the representative Western blot images, only one sample from each experimental group represented is included. On the other hand, to obtain the graph with the values of the densitometric quantification of the expression of CYP27A, the densitometric values from the six samples that conformed in each experimental group were included and analyzed, each of them corrected with the densitometric value of their respective β-actin used as a loading control. As can be seen in the published manuscript, in the description of the results obtained, the analyzed values of the densitometric quantification graph were considered, and, then, the conclusions of the article were made based on these data. Therefore, the scientific conclusions of the published article were not affected by the mentioned human error.

The correct image appears below. The authors state that the scientific conclusions are unaffected. This correction was approved by the Academic Editor. The original publication has also been updated.

**Figure 3:**
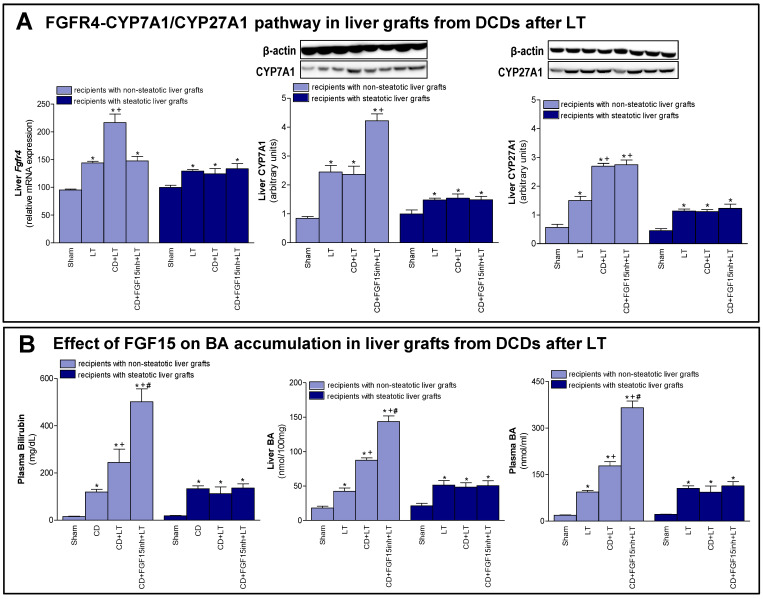
Effect of FGF15 on FGFR4-CYP7A1/CYP27A1 pathway and BA accumulation in LT with DCDs. (**A**) mRNA expression of Fgfr4 and protein expression of CYP7A1 and CYP27A1 in liver. (**B**) Bilirubin in plasma, and BAs in liver and plasma. * *p* < 0.05 vs. Sham; + *p* < 0.05 vs. LT; and # *p* < 0.05 vs. CD + LT.

**Figure 4:**
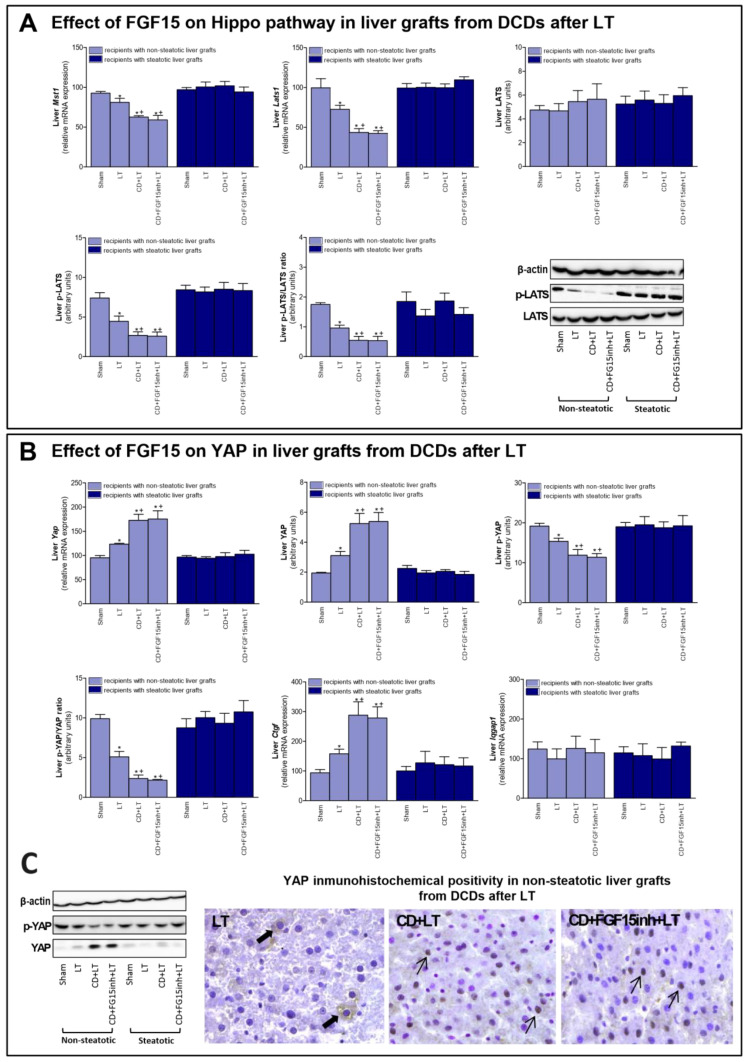
Effect of FGF15 on Hippo/YAP signaling pathway in LT with DCDs. (**A**) mRNA expression of Mst1, Lats1, and protein levels of LATS, p-LATS, and p-LATS/LATS ratio in liver. (**B**) mRNA levels of Yap, protein levels of YAP, p-YAP, p-YAP/YAP ratio; mRNA expression levels of Ctgf and Iqgap1 in liver. * *p* < 0.05 vs. Sham and + *p* < 0.05 vs. LT; # *p* < 0.05 vs. CD + LT. (**C**) Representative photomicrographs of YAP immunohistochemical positivity. Inmunostaining of cytoplasmatic YAP in hepatocyte (thick arrow) of LT group and YAP nuclear staining in CD + LT and CD + FGF15Inh + LT groups (thin arrow) (40×).

## References

[1] Avalos-de León C.G., Jiménez-Castro M.B., Cornide-Petronio M.E., Gulfo J., Rotondo F., Gracia-Sancho J., Casillas-Ramírez A., Peralta C. (2019). The Effect of Fibroblast Growth Factor 15 Signaling in Non-Steatotic and Steatotic Liver Transplantation from Cardiocirculatory Death. Cells.

